# Spatial Usage and Circadian Rhythm for Older Adults in the Community in Taiwan

**DOI:** 10.1093/geroni/igab046.2402

**Published:** 2021-12-17

**Authors:** Chih-Liang Wang, Ching-Ju Chiu

**Affiliations:** 1 National Cheng Kung University, Tainan City, Tainan, Taiwan (Republic of China); 2 National Cheng Kung University, Institute of Gerontology, College of Medicine, National Cheng Kung University, Tainan, Taiwan (Republic of China)

## Abstract

Objective measure of lifestyle of the older adults living in the community is void in the literature. To obtain both objective and subjective measurements to ascertain mobile and day and night lifestyle of older adults living in the community, and to build lifestyle model of older adults in the community by sociodemographic character. This study is a cross-sectional research. 200 over-50-year older adults who own smartphone and live in southern Taiwan were interviewed. Wrist accelerometers to detect behavioral circadian rhythm, GPS app in smartphone to survey mobility, and questionnaire to assess psychological and social status. Preliminary finding of six participants (2 men and 4 women) was analyzed. Data show that participants about 60 years old have large discrepancies in comparison with participants in their 50s: lower sleep efficiency (73 vs 83), earlier Most active 10 hour midpoint (11.48vs 14.13 hour), higher interdaily variability (0.84 vs 0.75), wake after sleep onset (100.39vs 47.78 minutes), and higher exercise frequency (4.33 vs 1.66 times per week). In addition, men have more chronic disease, bigger waistline (103.5 vs 77.5 cm), higher BMI (30 vs 22.5), lower middle to vigorous physical activity time (39 vs 79 minutes), and more total sleep time (356 vs 317 minutes). Age and sex seem to be significant factors determining lifestyle of older adults. Other sociodemographic parameters will be further analyzed.

